# Heterogeneous Immunolocalisation of Zinc Transporters ZIP6, ZIP10 and ZIP14 in Human Normo- and Asthenozoospermic Spermatozoa

**DOI:** 10.3390/cimb44080237

**Published:** 2022-07-31

**Authors:** Isidora Protic, Igor Golic, Snezana Vidakovic, Bato Korac, Aleksandra Korac

**Affiliations:** 1 Department of Medically Assisted Fertilisation of the Clinic for Gynecology and Obstetrics “Visegradska”, University Clinical Center of Serbia, 11000 Belgrade, Serbia; izidapro1@gmail.com (I.P.); drvidakovicsnezana@gmail.com (S.V.); 2 Faculty of Biology, University of Belgrade, 11000 Belgrade, Serbia; igor.golic@bio.bg.ac.rs; 3 Institute for Biological Research “Siniša Stanković”, National Institute of Republic of Serbia, University of Belgrade, 11000 Belgrade, Serbia; koracb@ibiss.bg.ac.rs

**Keywords:** zinc, ZIP, male infertility, asthenozoospermia, normozoospermia

## Abstract

Zinc (in the form of Zn^2+^) is necessary for male fertility. Both Zn^2+^ quantity and its localisation have been detected in seminal plasma and ejaculated spermatozoa, suggesting its active uptake via zinc import transporters (ZIPs). Immunofluorescence was used to characterise the expression and localisation of three distinct types of ZIP transporters in ejaculated spermatozoa of normo- and asthenozoospermic sperm samples. ZIP6, ZIP10 and ZIP14 showed heterogeneous sperm cell expression and different compartmental distribution. In both types of sperm samples, ZIP6 and ZIP14 were predominantly localised in the sperm head, while ZIP10 was found along the sperm tail. Compartmental localisation of ZIPs in asthenozoospermia was not changed. However, regarding sub-compartmental localisation in sperm head regions, for ZIP6 asthenozoospermia only decreased its acorn/crescent-like pattern. In contrast, ZIP14 immunostaining was altered in favour of crescent-like, as opposed to acorn-like and acorn/crescent-like patterns. The specific ZIPs localisation may reflect their different roles in sperm cell integrity and motility and may change over time. This is the first report of their specific compartmental and sub-compartmental localisation in ejaculated human sperm cells. Further research will lead to a greater understanding of the roles of ZIPs in sperm cell biology, which could positively influence procedures for human infertility therapy.

## 1. Introduction

Zinc (Zn^2+^) is the second-most abundant essential trace element found in the human body [1]. Biological roles of Zn^2+^ include signalling, enzymatic activities, regulation of normal growth and sexual maturation [2]. Regular intake of Zn^2+^ is important for its constant availability, since no specific Zn^2+^ depot is known to exist [3]. Although it is required by every cell, the subcellular Zn^2+^ distribution and/or content depends on cell type and function.

There is significant evidence indicating that Zn^2+^ has different roles in the male reproductive system; regulation of sperm structure and function, spermatogenesis (from a developing germ cell to a spermatozoon), epididymal sperm maturation, sperm cell motility, sperm interactions with the female reproductive tract, capacitation, fertilisation and embryo development [2,4].

From several perspectives, Zn^2+^ plays a key role in spermatogenesis. The concentration of Zn^2+^ in the testes increases during the early spermatogenesis period to regulate spermatogonial proliferation and to protect germ cells against damage during meiosis [5]. The concentration of Zn^2+^ is relatively high in developing spermatocytes due to its requirement for DNA condensation, meiosis and its general accumulation in the cell [5,6]. During the final stages of differentiation Zn^2+^ readily enters spermatid cells facilitating DNA condensation [6]. Additional Zn^2+^ incorporation into the nucleus of ejaculated sperm is believed to have a protective function in terms of sperm chromatin decondensation, sperm motility, metabolic inhibition, membrane stabilisation and antioxidant activity [2,4].

At the end of spermatogenesis, Zn^2+^ is highly concentrated in the sperm flagellum bound to sulphydryl groups of outer dense fibre (ODFs) protein cysteine groups providing protection from premature oxidation [7]. High concentrations of Zn^2+^ have also been found in the acrosome. Zn^2+^ can provide energy for spermatozoal motion via lipid catabolism in the sperm midpiece [8]. Zn^2+^ also associates with sperm membranes, causing interactions with lipoproteins, thereby providing membrane stabilization [9]. In addition to the many roles of Zn^2+^ for the proper development of germ cells, Zn^2+^ toxicity must also be considered. The human body is exposed daily to metals through diet, environment and/or dietary supplementation [10]. Overexposure to Zn^2+^ may affect sperm quality, especially in treatment of idiopathic male infertility [11], which is why Zn^2+^ transporters are essential in regulating cellular Zn^2+^ requirements.

Zn^2+^ homeostasis is accomplished by its uptake, distribution, accumulation and efflux through biological membranes via two families of zinc transporter proteins [12]. These are the zinc-importer (ZIP; Zrt-, Irt-like) family of proteins that transport Zn^2+^ into the cytosol and the zinc transporter (Zn^2+^ T) family proteins transporting Zn^2+^ out of the cytosol. Through genomic sequencing 14 members of ZIP and 10 members of Zn^2+^ T families have been identified [2]. Human ZIP members are classified into I, II, LIV-1 and gufA subfamilies [13].

Although rarely reported in literature, the expression and function of Zn^2+^ transporters in male germ cells suggest that their role is for controlling Zn^2+^ influx [4]. The presence of various Zip proteins in different cell types during spermatogenesis in mice (ZIP1, ZIP5, ZIP6, ZIP8, ZIP10 and ZIP14) and humans (ZIP1, ZIP5, ZIP6, ZIP8) suggests their role in Zn^2+^ import in a specific stage of sperm cell formation [4,6]. However, the data concerning their overall contribution remains to be elucidated [4]. In a mice study by Croxford and associates, ZIP6 was expressed in round and elongating spermatids, ZIP10 was expressed in primary spermatocytes and round spermatids, while ZIP14 was present in the spermatogonia [6]. Regarding human zinc transporters, ZIP10 has not been studied in human testicular samples. In a human study, Foresta and associates showed ZIP6 presence during spermatogenesis at all cell stages, including testicular spermatozoa, while no ZIP14 expression was found [4]. In addition to having examined ZIP12 immunolocalisation in human testis, Zhu and associates [14] found its presence in the sperm head and midpiece of spermatozoa.

Without a doubt, Zn^2+^ is necessary for mammalian male fertility and its scarcity could affect sperm quality. The presence of certain ZIP transporters in the testes has different roles during the process of spermatogenesis, depending on the germinative cell stages. Despite their roles, the dynamics of Zn^2+^ transport remains unclear [4]. Both Zn^2+^ quantity and its localisation have been detected in seminal plasma and ejaculated spermatozoa [15,16], suggesting its active uptake via ZIP transporters.

By careful examination of UniProt and available studies of ZIPs in human testis and ejaculated sperm indicating specific expression patterns of ZIPs and physiological role of Zn^2+^ for all of three spermatozoa compartments (head, midpiece and tail), we chose to study ZIP6, ZIP10 and ZIP14 [1,2,3,4,5,6,7,8,9,10,11,12,13,14,15,16]. We used immunofluorescence to characterise their immunoexpression and localisation in ejaculated spermatozoa in normo- and asthenozoospermic samples.

## 2. Materials and Methods

### 2.1. Collection and Preparation of Samples

Immunofluorescence procedures were performed on sperm that was collected after obtaining written consent from males who were undergoing intrauterine insemination (IUI) fertility treatment with their respective female partners at the Department of Medically Assisted Fertilisation of the Clinic for Gynecology and Obstetrics “Visegradska”, University Clinical Center of Serbia, Belgrade, Serbia.

A total of 50 semen samples from 50 individuals were collected in sterile containers after 3–5 days of sexual abstinence. After liquefaction at room temperature, ejaculates were deemed normozoospermic or asthenozoospermic after standard assessments of semen according to World Health Organization (WHO) recommendations 2021 [17]. After excluding patients with recognizable risk factors for male infertility or previously reported medical history, in order to evaluate cell heterogeneity in the sperm population per patient, 4 samples were chosen. Two normozoospermic and two asthenozoospermic healthy patients age between 31 and 36, without children, had a total average body mass index (BMI) of 26.83. Semen analysis showed average volume for normo- and asthenozoospermia to be 3.75 mL, with an average sperm count for normo- (170 × 10^6^/mL) and asthenozoospermia (97.5 × 10^6^/mL). The normozoospermic semen samples had the progressive motility of 50%, while the motility of asthenozoospermic samples was below that value. For both types of samples, normal morphology exceeded 30%.

To remove non-spermatic cells, leukocytes and immobile sperm samples were purified by a modified density gradient centrifugation method. Pre-prepared SpermGrad 90% and 45% stock solutions (Vitrolife^®^, Göteborg, Sweden) were used for centrifugation. We used the general protocol for density gradient centrifugation method for SpermGrad according to the instructions of the manufacturer in order to obtain a pure resuspended pellet. To purify the sperm sample, the first centrifugation was performed for 20 min at 300 g, followed by two centrifugations at 300 g for 15 min, with one rinsing between each centrifugation using 5 mL of 0.9% saline. The centrifuged pellet was resuspended in 0.5 mL of 0.9% saline for further use [18].

### 2.2. Immunofluorescence

For immunofluorescence sperm smears on microscope slides were fixed for 15 min in methanol and then air-dried. The sperm smears were additionally fixed and permeabilised using acetone for 5 min, with further permeabilisation using 0.3% Triton X-100 in TBS (tris-buffered saline) for 10 min. After blocking with 0.1% BSA (bovine serum albumin), 3% NGS (normal goat serum), 0.2% Triton, and 0.05% Tween-20 in TBS for 30 min, smears were incubated with the primary antibodies: ZIP6 (1:50, PA5-21071, Invitrogen, Carlsbad, CA, USA), ZIP10 (1:50, PA5-21064, Invitrogen) and ZIP14 (1:50, PA5-21077, Invitrogen) in the same blocking solution overnight at 4 °C. The following day, after being rinsed three times in TBS-T (tris-buffered saline Tween 20), sperm smears were labelled with Alexa Fluor 488 secondary antibody (1:400, A-11029, Invitrogen) in blocking solution one hour at room temperature. Thereafter, the slides were rinsed twice in TBS-T and once in TBS. Counterstaining was performed with nuclear stain Sytox Orange (1:1000, S11368, Life Technologies, Gaithersburg, MD, USA) for 5 min. After this final step, the slides were rinsed in TBS and mounted with Mowiol [19]. All chemicals used in this study were purchased from Sigma-Aldrich (Steinheim, Germany), if not otherwise stated.

Images were obtained with Leica TCS SP5 II confocal microscope (Leica Microsystems, Wetzlar, Germany) in sequential mode to avoid crosstalk between channels. The smears were excited with a 488 nm laser. Nuclei were detected using a 543 nm laser and blue false-coloured for clear distinction of the green channel. The specificity of immunofluorescence was confirmed by omission of primary antibodies. Negative controls were performed in parallel to investigate non-specific staining.

The number of immunopositive sperm cells, their compartments (head, midpiece and tail), head sub-compartments of anterior region (acorn-like, crescent-like, acorn/crescent-like patterns) and posterior region were counted per spermatozoon, respectively, using multi-point tool in ImageJ software (NIH, Bethesda, MD, USA) and expressed as a percentage. For each type of sample, six randomly selected field areas were analysed. Every field area had more than 100 spermatozoa.

### 2.3. Statistics

Statistical analyses were performed using Prism 8, version 8.4.3. (GraphPad Software, San Diego, CA, USA). The non-parametric Kruskal–Wallis test was used to test within-group comparisons (between compartments). If the F test indicated an overall difference, Dunn’s multiple comparison test was applied to evaluate the significance of the difference. The Mann–Whitney U test was used to test overall immunopositivity of sperm samples and the significance of differences between the means of the same sperm head sub-compartment of two groups. Statistical significance was considered at *p* ≤ 0.05.

## 3. Results

Immunofluorescent detection of ZIP6, ZIP10 and ZIP14 was performed in both normozoospermic and asthenozoospermic samples. Washed and processed sperm were obtained via the modified sperm gradient method. ZIP6, ZIP10 and ZIP14 showed heterogeneous expression in sperm cells. In addition, heterogeneity was observed in cell compartments and sperm head sub-compartments of the anterior region (acorn-like, crescent-like, acorn/crescent-like patterns) and the posterior region.

### 3.1. ZIP6

Overall immunopositivity for ZIP6 decreased in asthenozoospermia (30.28% ± 4.14) compared to normozoospermia (79.05% ± 2.08, *p* ≤ 0.001).

**Figure 1:**
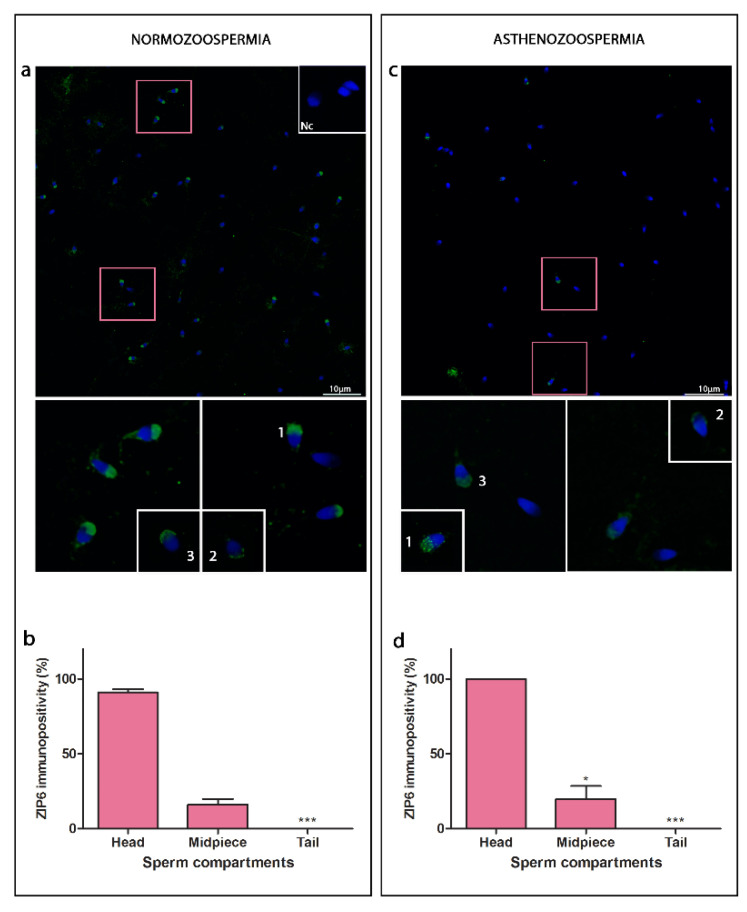
Immunofluorescence detection of ZIP6 transporter in normozoospermic (**a**) and asthenozoospermic (**c**) sperm samples using a confocal laser scanning microscope. Sub-compartments of anterior head region: acorn-like (**1**), crescent-like (**2**), acorn/crescent-like (**3**) patterns and posterior head region. Nc—negative control. Scale bar = 10 µm. Average of total number of spermatozoa per ejaculate before processing was in normo- (625 × 10^6^/vol) and asthenozoospermia (367.5 × 10^6^/vol); For each type of sample, six randomly selected field areas were analysed (>100 cells). Relative immunopositivity of ZIP6 among the compartments in the normozoospermic (**b**) and asthenozoospermic (**d**) sperm samples. * in comparison to head, * *p* ≤ 0.05, *** *p* ≤ 0.001.

In normozoospermic samples ZIP6 was predominantly localised in the sperm head (Figure 1a,b, Table 1). In the anterior head region ZIP6 formed clusters with specific patterns, prominent acorn-like, less present crescent-like and combination of the two. Immunostaining was also observed in a posterior region of the head. ZIP6 immunostaining was also noticed around the head of some sperm cells. In the midpiece, ZIP6 was localised in a few sperm cells, while no immunostaining was present along the tail.

In asthenozoospermic samples (Figure 1c,d, Table 1), ZIP6 head sub-compartmental immunolocalisation remained unchanged, except for a decrease in acorn/crescent-like patterns in the anterior region. There was no difference in ZIP6 expression in the midpiece and tail compared to normozoospermia.

### 3.2. ZIP10

**Figure 2:**
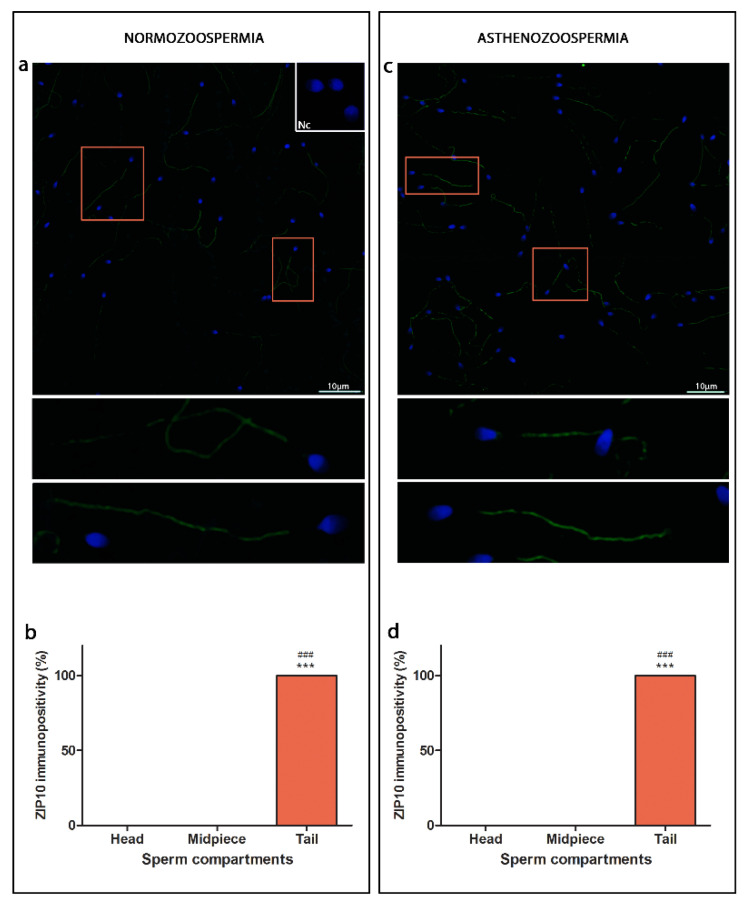
Immunofluorescence detection of ZIP10 transporter in normozoospermic (**a**) and asthenozoospermic (**c**) sperm samples using a confocal laser scanning microscope. Sub-compartments of anterior head region: acorn-like (**1**), crescent-like (**2**), acorn/crescent-like (**3**), wreath-like (**4**) patterns and posterior head region. Nc—negative control. Scale bar = 10 µm. Average of total number of spermatozoa per ejaculate before processing was in normo- (625 × 10^6^/vol) and asthenozoospermia (367.5 × 10^6^/vol); For each type of sample, six randomly selected field areas were analysed (>100 cells). Relative immunopositivity of ZIP10 among the compartments in the normozoospermic (**b**) and asthenozoospermic (**d**) sperm samples. * in comparison to head, ^#^ in comparison to midpiece, *** *p* ≤ 0.001, ^###^ *p* ≤ 0.001.

Overall immunopositivity for ZIP10 in both the normo- and asthenozoospermic samples was similar (Figure 2). ZIP10 immunostaining was mainly located along the sperm tails, while absent in the head and midpiece.

### 3.3. ZIP14

Overall immunopositivity for ZIP14 decreased in asthenozoospermia (39.00% ± 8.77) compared to normozoospermia (60.01% ± 7.30).

**Figure 3:**
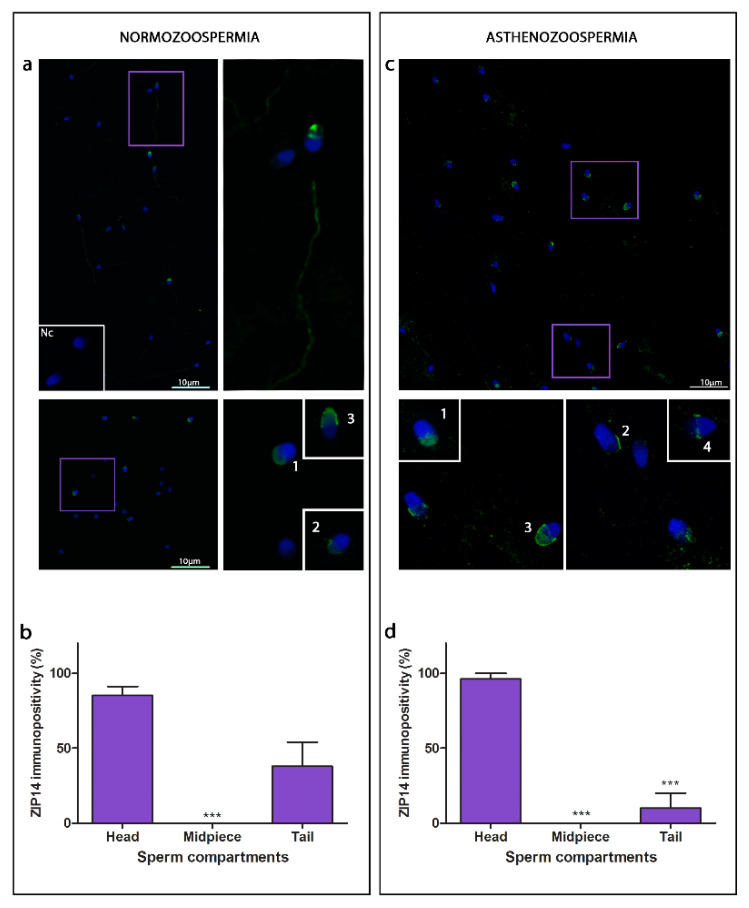
Immunofluorescence detection of ZIP14 transporter in normozoospermic (**a**) and asthenozoospermic (**c**) sperm samples using a confocal laser scanning microscope. Nc—negative control. Scale bar = 10 µm. Average of total number of spermatozoa per ejaculate before processing was in normo- (625 × 10^6^/vol) and asthenozoospermia (367.5 × 10^6^/vol); For each type of sample, six randomly selected field areas were analysed (>100 cells). Relative immunopositivity of ZIP14 among the compartments in the normozoospermic (**b**) and asthenozoospermic (**d**) sperm samples. * in comparison to head, *** *p* ≤ 0.001.

ZIP14 immunostaining was mainly found in both the head and the tail (Figure 3, Table 2). Asthenozoospermia affected head sub-compartments the most, changing the specific immunostaining in favour of crescent-like, opposite to acorn-like and acorn/crescent-like patterns. In normozoospermic samples, ZIP14 immunostaining around the head was observed in a few sperm cells. In asthenozoospermia, ZIP14 expressed a wreath-like immunostaining pattern localised in the equatorial region of few sperm heads (Figure 3c). In both normo- and asthenozoospermia, ZIP14 immunostaining was observed in tails but not detected in the midpieces.

## 4. Discussion

Using specific immunofluorescent labelling and confocal microscopy techniques, we demonstrated expression and immunolocalisation of ZIP6, ZIP10 and ZIP14 in ejaculated human sperm cells classified as normo- and asthenozoospermic. We found that sperm cells in both examined groups expressed all three ZIPs. To our knowledge, this is the first study showing their specific compartmental and sub-compartmental localisation in ejaculated human sperm cells.

### 4.1. ZIPs Are Heterogeneously Expressed in Sperm Cells; Lower Level of Overall Immunostaining of ZIP6 Is Found in Asthenozoospermia

ZIP1, ZIP5, ZIP6, ZIP8 and ZIP12 expression has been examined mainly in human testes [4,14]. Foresta and colleagues reported the presence of ZIP6 transporters in all cell stages of spermatogenesis. Regarding ZIP10 and ZIP14, their presence was not assessed (ZIP10) or found (ZIP14) in humans [4]. Only few studies concerning ejaculated human sperm cells found ZIP1, ZIP5, ZIP6 and ZIP8 [4]. In view of the high-level expression of the ZIPs during spermatogenesis and their limited presence in ejaculated sperm, the key question concerns their origin. On one hand, it is clear that some of the ZIPs could be retained after spermatogenesis. On the other hand, it has been shown that boar sperm cells can insert vesicles originating from the seminal plasma [20]. In addition, Murdica and colleagues reported that after ejaculation, human sperm cells were still receptive and are able to take up exosomes from seminal plasma [21]. The previously reported presence of Zn^2+^ transporters in different regions of the epididymis supports this [22]. It is interesting that Park and colleagues showed that sperm could acquire Ca^2+^ signalling machinery necessary for motility through the fusion of semen exovesicles with the midpiece [23]. This may explain the heterogeneity in the number of immunopositive sperm found in this study. Significant reduction in seminal plasma zinc concentrations was observed in asthenozoospermic men [24], which is why localization of ZIPs is important, since the lack of Zn^2+^ could possibly be overcome by Zn^2+^ supplementation and intake via transporters in the spermatozoa.

More additional experiments are needed to elucidate the underlying mechanism(s). This may also provide an answer to whether the observed lower overall ZIP6 immunopositivity in asthenozoospermia was inherited or acquired.

### 4.2. ZIPs Show Different Compartmental Localisation, ZIP6 and ZIP14 Are Found in the Head, While ZIP10 Are Located Entirely along the Sperm Cell Tail

ZIP6, ZIP10 and ZIP14 exhibit specific compartmentalization, with respect to sperm head, midpiece and tail. In both types of samples, ZIP6 and ZIP14 were predominantly localised in the sperm head, while ZIP10 was found in sperm tails. This localisation was in accordance with an autometallographic study showing Zn^2+^ localisation in all three sperm cell compartments in human ejaculated sperm smears [16]. It is interesting that Zhu and colleagues found ZIP12 in the midpiece and head of testicular human spermatozoa which suggest its early compartmentalisation [14]. In line with this, our results suggest that different ZIPs could contribute to different spatial and time-dependent Zn^2+^ import activities.

The overall lower expression of ZIP6 in asthenozoospermic samples implies that ZIP6 could be a marker of sperm maturity. Kerns and colleagues showed different zinc signatures (the zinc code) among sperm cells and their correlation with sperm maturity and fertilisation success. Hence, the observed heterogenous expression of ZIPs could be a consequence of previously reported variable inter-cell Zn^2+^ nuclear concentrations and the zinc code [25,26].

### 4.3. Specific Head Sub-Compartment Localisation of ZIP6 and ZIP14

We noted ZIP-specific sub-comparmental immunolocalisation patterns in the anterior (acorn, crescent and acorn/crescent) and posterior region of the head. Using atomic absorption spectrometry, Henkel and colleagues found that sperm heads contained only 6.5% of the total amount of Zn^2+^ found in human spermatozoa [27]. It is interesting that we found positive immunostaining of the sperm head for both ZIP6 and ZIP14 despite such a surprisingly low Zn^2+^ concentration in the head. In the anterior head region ZIP6 remained unchanged, except for the sub-compartmental localisation of acorn/crescent like pattern that was diminished in asthenozoospermia. Noted different sub-compartmental expression of ZIP14 between the two sperm sample groups remains unclear. Zn^2+^ plays a role in stabilisation of nuclear chromatin, particularly in sperm cells. Sperm cells import additional Zn^2+^ from seminal fluid and incorporate it into the nucleus at ejaculation [28]. Since chromatin inactivity in ejaculated spermatozoa [29] is heterogeneous as well as the nuclear Zn^2+^ concentration [25], it could be speculated that the effect of asthenozoospermia on spermatozoa is not only characterised by defective motility, but also by their relative nuclear integrity/stability [30,31].

ZIP6’s presence in the posterior head region of the sperm cells was present in both sperm samples. ZIP14, however, was completely absent. Since the human sperm nuclear envelope lacks nuclear pores, except in a very limited region between the posterior ring and the basal plate [32], everything points towards maintaining chromatin stability in ejaculated sperm cells.

We also detected positive immunostaining for ZIP6 and ZIP14 transporters around the sperm head of some cells within both types of sperm samples. Human seminal plasma contains exosomes that have been shown to play important roles in increasing sperm motility and delaying acrosomal reaction [20]. Studies on boar seminal plasma exosomes showed a prolonged sperm motility time [20]. Even though we used washed samples, it may be possible that some of the vesicles remained with a specific protein cargo of transporters, whose role may be in ensuring Zn^2+^ presence for future fertilisation [21]. This finding is very intriguing and further studies are needed for clarification.

We found ZIP6 in the midpiece in some cells. The precise role of ZIP transporters in the midpiece remains unknown. Midpiece is known to have a role in providing Zn^2+^ for internal cell storage and potential use in the mitochondria [25,26]. Some studies reported that Zn^2+^ stabilises head–tail connection and inhibits their detachment [33].

The molecular basis of Zn^2+^ metabolism is also associated with sperm motility. ZIP10 was mainly localised in the sperm tail in both types of sperm samples. As for ZIP14, tail immunopositivity was also present, but reduced in asthenozoospermic samples. Some researchers found that highly motile sperm contained a significantly lower Zn^2+^ content compared to slow or non-motile spermatozoa [4], while others found that when the Zn^2+^ concentration in the flagella was high, sperm motility and velocity were low [27]. The latter suggested that decreased stiffness of the ODFs, a result of excessive Zn^2+^ in the flagella, might be a cause of asthenozoospermia [27]. In addition, the absence of ZIP6 in the sperm tail in both types of sperm samples suggested that it played no role in motility. Judging by the contradictory results with respect of the role of Zn^2+^ in the tail of spermatozoa, further research is needed to uncover its precise role. Additionally, tail immunostaining of ZIP10 and ZIP14 still needs further clarification.

In our study, the small sample size may be considered as a limitation. Nevertheless, to our knowledge, this is the first study pointing out the real differences between individual sperm cells at the cellular and molecular level. In the era of a more personalised approach in medicine, these data speak for the individual sperm cell repertoire from which only one can fertilise the oocyte. Hence, even the slightest changes in one sperm cell could be a specific molecular signature for potential fertilisation success.

Understanding heterogeneity among sperm cells in normozoospermic and asthenozoospermic samples with respect to regarding compartmental and sub-compartmental immunolocalisation of ZIPs will provide novel insight into the biology of spermatozoa. However, a further comprehensive functional study would be valuable, and our future study will be directed towards it.

## 5. Conclusions

Heterogeneity among sperm cells in normozoospermic and asthenozoospermic samples with respect to compartmental and sub-compartmental immunolocalisation of ZIPs is evident. ZIP6, ZIP10 and ZIP14 showed heterogeneous sperm cell expression and different compartmental distribution. In both types of sperm samples, ZIP6 and ZIP14 were predominantly localised in the sperm head, while ZIP10 was found along the sperm tail. While compartmental localisation of ZIPs in asthenozoospermia was not changed, ZIP6 and ZIP14 showed differences in sub-compartmental localisation in sperm head regions. This specific ZIPs localisation may reflect their different roles in sperm cell integrity and motility and may change over time. Many questions regarding Zn^2+^ transport mechanisms remain, and they need to be addressed. Additional research is needed to better understand ZIPs’ roles in sperm cell biology which could positively influence procedures for human infertility therapy.

## Figures and Tables

**Table 1:** Percentage of ZIP6 immunolocalisation in the head sub-compartments of sperm cells.

	Head of the Spermatozoa			
	Anterior Head Region (%)			Posterior Head Region (%)
	Acorn-Like Pattern	Crescent-Like Pattern	Acorn/Crescent-Like Pattern	
Normozoospermia	64.61 ± 6.18	19.81 ± 4.09	12.68 ± 2.90	33.45 ± 7.64
Asthenozoospermia	71.32 ± 9.95	21.54 ± 8.29	1.52 ± 1.52 **	56.49 ± 14.84

Average of total number of spermatozoa per ejaculate before processing was in normo- (625 × 10^6^/vol) and asthenozoospermia (367.5 × 10^6^/vol); For each type of sample, six randomly selected field areas were analysed (>100 cells). The results are the means ± SEM; * compared to normozoospermia. ** *p* ≤ 0.01.

**Table 2:** Percentage of ZIP14 immunolocalisation in the head sub-compartments of sperm cells.

	Head of the Spermatozoa			
	Anterior Head Region (%)			Posterior Head Region (%)
	Acorn-Like pattern	Crescent-Like Pattern	Acorn/Crescent like-Pattern	
Normozoospermia	68.08 ± 2.58	18.50 ± 2.91	14.66 ± 3.22	0.00
Asthenozoospermia	17.57 ± 6.57 ***	67.63 ± 9.36 ***	2.38 ± 2.38 **	0.00

Average of total number of spermatozoa per ejaculate before processing was in normo- (625 × 10^6^/vol) and asthenozoospermia (367.5 × 10^6^/vol); For each type of sample, six randomly selected field areas were analysed (>100 cells). The results are the means ± SEM; * compared to normozoospermia. ** *p* ≤ 0.01, *** *p* < 0.001.

## Data Availability

Not appliable.

## References

[1] Kambe T., Nishito Y., Fukue K., Collins J.F. (2017). Chapter 23—Zinc transporters in health and disease. Molecular, Genetic, and Nutritional Aspects of Major and Trace Minerals.

[2] Kerns K., Zigo M., Sutovsky P. (2018). Zinc: A necessary ion for mammalian sperm fertilization competency. Int. J. Mol. Sci..

[3] Scott B.J., Bradwell A.R. (1983). Identification of the serum binding proteins for iron, zinc, cadmium, nickel, and calcium. Clin. Chem..

[4] Foresta C., Garolla A., Cosci I., Menegazzo M., Ferigo M., Gandin V., De Toni L. (2014). Role of zinc trafficking in male fertility: From germ to sperm. Hum. Reprod..

[5] Vickram S., Rohini K., Srinivasan S., Veenakumari D.N., Archana K., Anbarasu K., Jeyanthi P., Thanigaivel S., Gulothungan G., Rajendiran N. (2021). Role of zinc (Zn) in human reproduction: A journey from initial spermatogenesis to childbirth. Int. J. Mol. Sci..

[6] Croxford T.P., McCormick N.H., Kelleher S.L. (2011). Moderate zinc deficiency reduces testicular Zip6 and Zip10 abundance and impairs spermatogenesis in mice. J. Nutr..

[7] Lee S.R. (2018). Critical role of zinc as either an antioxidant or a prooxidant in cellular systems. Oxidative Med. Cell. Longev..

[8] Fallah A., Mohammad-Hasani A., Colagar A.H. (2018). Zinc is an essential element for male fertility: A review of Zn roles in men’s health, germination, sperm quality, and fertilization. J. Reprod. Infertil..

[9] Bettger W.J., O’Dell B.L. (1981). A critical physiological role of zinc in the structure and function of biomembranes. Life Sci..

[10] Xu S., Wu Y., Chen Y., Lu W., Wang Y.X., Gao B., Zhang J. (2022). Environmental metal exposure, seminal plasma metabolome and semen quality: Evidence from Chinese reproductive-aged men. Sci. Total Environ..

[11] Garolla A., Petre G.C., Francini-Pesenti F., De Toni L., Vitagliano A., Di Nisio A., Grande G., Foresta C. (2022). Systematic Review and Critical Analysis on Dietary Supplements for Male Infertility: From a Blend of Ingredients to a Rationale Strategy. Front. Endocrinol..

[12] Kambe T., Tsuji T., Hashimoto A., Itsumura N. (2015). The physiological, biochemical, and molecular roles of zinc transporters in zinc homeostasis and metabolism. Physiol. Rev..

[13] Kambe T., Yamaguchi-Iwai Y., Sasaki R., Nagao M. (2004). Overview of mammalian zinc transporters. Cell. Mol. Life Sci..

[14] Zhu X., Yu C., Wu W., Shi L., Jiang C., Wang L., Ding Z., Liu Y. (2022). Zinc transporter ZIP12 maintains zinc homeostasis and protects spermatogonia from oxidative stress during spermatogenesis. Reprod. Biol. Endocrinol..

[15] Atig F., Raffa M., Habib B.A., Kerkeni A., Saad A., Ajina M. (2012). Impact of seminal trace element and glutathione levels on semen quality of Tunisian infertile men. BMC Urol..

[16] Stoltenberg M., Sørensen M.B., Danscher G. (1997). Histochemical demonstration of zinc ions in ejaculated human semen. Int. J. Androl..

[17] WHO Laboratory Manual for the Examination and Processing of Human Semen. https://www.who.int/publications-detail-redirect/9789240030787.

[18] Protic I., Golic I., Aleksic M., Vidakovic S., Korac B., Korac A. (2022). Presence of acetylated α-tubulin in human sperm nuclei: A contributor to sperm heterogeneity. Med. Hypotheses.

[19] Golić I., Aleksić M., Lazarević A., Bogdanović M., Jonić S., Korać A. (2016). Methods for studying the localization of mitochondrial complexes III and IV by immunofluorescent and immunogold microscopy. Arch. Biol. Sci..

[20] Du J., Shen J., Wang Y., Pan C., Pang W., Diao H., Dong W. (2016). Boar seminal plasma exosomes maintain sperm function by infiltrating into the sperm membrane. Oncotarget.

[21] Murdica V., Giacomini E., Alteri A., Bartolacci A., Cermisoni G.C., Zarovni N., Papaleo E., Montorsi F., Salonia A., Viganò P. (2019). Seminal plasma of men with severe asthenozoospermia contain exosomes that affect spermatozoa motility and capacitation. Fertil. Steril..

[22] Bedwal R.S., Bahuguna A. (1994). Zinc, copper and selenium in reproduction. Experientia.

[23] Park K.H., Kim B.J., Kang J., Nam T.S., Lim J.M., Kim H.T., Park J.K., Kim Y.G., Chae S.-W., Kim U.-H. (2011). Ca^2+^ signaling tools acquired from prostasomes are required for progesterone-induced sperm motility. Sci. Signal..

[24] Taravati A., Tohidi F. (2016). Association between seminal plasma zinc level and asthenozoospermia: A meta-analysis study. Andrologia.

[25] Kvist U., Björndahl L., Roomans G.M., Lindholmer C. (1985). Nuclear zinc in human epididymal and ejaculated spermatozoa. Acta Physiol. Scand..

[26] Kerns K., Zigo M., Drobnis E.Z., Sutovsky M., Sutovsky P. (2018). Zinc ion flux during mammalian sperm capacitation. Nat. Commun..

[27] Henkel R., Bittner J., Weber R., Hüther F., Miska W. (1999). Relevance of zinc in human sperm flagella and its relation to motility. Fertil. Steril..

[28] Bjorndahl L., Kjellberg S., Roomans G.M., Kvist U. (1986). The human sperm nucleus takes up zinc at ejaculation. Int. J. Androl..

[29] Mirnamniha M., Faroughi F., Tahmasbpour E., Ebrahimi P., Harchegani A.B. (2019). An overview on role of some trace elements in human reproductive health, sperm function and fertilization process. Rev. Environ. Health.

[30] Colleu D., Lescoat D., Boujard D., Le Lannou D. (1988). Human spermatozoal nuclear maturity in normozoospermia and asthenozoospermia. Arch. Androl..

[31] Kumar D., Kalthur G., Mascarenhas C., Kumar P., Adiga S.K. (2011). Ejaculate fractions of asthenozoospermic and teratozoospermic patients have differences in the sperm DNA integrity. Andrologia.

[32] Mortimer D. (2018). The functional anatomy of the human spermatozoon: Relating ultrastructure and function. Mol. Hum. Reprod..

[33] Björndahl L., Kvist U. (1982). Importance of zinc for human sperm head-tail connection. Acta Physiol. Scand..

